# Activation of cellular antioxidative stress and migration activities by purified components from immortalized stem cells from human exfoliated deciduous teeth

**DOI:** 10.1038/s41598-024-66213-8

**Published:** 2024-07-03

**Authors:** Yujing Shu, Masato Otake, Yasuhiro Seta, Keigo Hori, Akiko Kuramochi, Yoshio Ohba, Yuji Teramura

**Affiliations:** 1 U-Factor Co., Ltd, 1F, ESCALIER Rokubancho, 7-11, Rokubancho, Chiyoda, Tokyo, 102-0085 Japan; 2 Hitonowa Medical, K.PLAZA 2F, 1-7 Rokubancho, Chiyoda, Tokyo, 102-0085 Japan; 3 Cellular and Molecular Biotechnology Research Institute (CMB), National Institute of Advanced Industrial Science and Technology (AIST), AIST Tsukuba Central 5, 1-1-1 Higashi, Tsukuba, Ibaraki 305-8565 Japan; 4 Department of Immunology, Genetics and Pathology (IGP), Uppsala University, Dag Hammarskjölds Väg 20, 751 85 Uppsala, Sweden; 5 Master’s/Doctoral Program in Life Science Innovation (T-LSI), University of Tsukuba, 1-1-1 Tennodai, Tsukuba, Ibaraki 305-8577 Japan

**Keywords:** Regenerative therapy, Cytokines, Stem cells from human exfoliated deciduous teeth (SHEDs), Conditioned media, Immortalization, Stem cells, Mesenchymal stem cells

## Abstract

Although stem cell-based regenerative medicine has been extensively studied, it remains difficult to reconstruct three dimensional tissues and organs in combination with vascular systems in vitro. One clinically successful therapy is transplantation of mesenchymal stem cells (MSC) into patients with graft versus host disease. However, transplanted cells are immediately damaged and destroyed because of innate immune reactions provoked by thrombogenic inflammation, and patients need to take immunosuppressive drugs for the immunological regulation of allogeneic cells. This reduces the benefits of stem cell transplantation. Therefore, alternative therapies are more realistic options for clinical use. In this study, we aimed to take advantage of the therapeutic efficacy of MSC and use multiple cytokines released from MSC, that is, stem cells from human exfoliated deciduous teeth (SHEDs). Here, we purified components from conditioned media of immortalized SHED (IM-SHED-CM) and evaluated the activities of intracellular dehydrogenase, cell migration, and antioxidative stress by studying the cells. The immortalization of SHED could make the stable supply of CM possible. We found that the fractionated component of 50–100 kD from IM-SHED-CM had higher efficacy than the original IM-SHED-CM in terms of intracellular dehydrogenase and cell migration in which intracellular signal transduction was activated via receptor tyrosine kinases, and the glutathione peroxidase and reductase system was highly active. Although antioxidative stress activities in the fractionated component of 50–100 kD had slightly lower than that of original IM-SHE-CM, the fraction still had the activity. Thus, the use of fractionated components of 50–100 kD from IM-SHED-CM could be an alternative choice for MSC transplantation because the purified components from CM could maintain the effect of cytokines from SHED.

## Introduction

Stem cell-based therapies have been extensively studied in regenerative medicine^1^. Because organ and cell transplantations derived from human donors suffer from serious donor shortages, stem cells could be ideal resources for clinical therapy. However, stem cell-based therapy is yet to be considered as clinical therapy because it is difficult to make three-dimensional organs and tissues in vitro together with vascular systems for connection with recipients after transplantation. In addition, the safety issue of tumor formation, that is, teratoma formation, remains unclear^2,3^. However, some successful clinical cases of stem cell-based therapies have been reported^4,5^. Bone marrow-derived mesenchymal stem cells (MSC) have been used to treat patients with graft versus host disease, and most clinical studies have shown some efficacy, although response rates have varied. MSCs are generally safe and well-tolerated because the cells have a modulation of the local immune response with a down-regulation of the innate and adaptive immunity, leading to the immune privilege^6,7^. MSCs have also been studied for stroke treatment and their clinical outcomes have been encouraging. In addition, other types of MSCs, dental pulp-derived stem cells such as stem cells from human exfoliated deciduous teeth (SHEDs) and human adult dental pulp stem cells, are potent candidates for stem cell-based therapies^8,9^.

However, transplanted cells are immediately damaged or destroyed owing to hemoincompatibility caused by tissue factor when infused into the bloodstream of the patient, resulting in the activation of coagulation cascade and complement activation, followed by cell destruction^10,11^. This issue applies not only to stem cells but also to donor-derived cells, such as human pancreatic islets and hepatocytes^12,13^. Our group found that cell destruction during the early stage of transplantation was caused by innate immune reactions and termed this phenomenon instant blood-mediated inflammatory reaction or thromboinflammation^14–16^. One approach to avoid an immediate destructive response is cell surface modification with biocompatible polymers^17–19^, which has been studied clinically. Therefore, the clinical use of stem cells in patients needs to be carefully considered because of their low efficacy and hemoincompatibility. Stem cell transplantation is allogeneic; therefore, immune rejection must be regulated by immunosuppressive drugs. This reduces the benefits of stem cell transplantation. Therefore, regenerative therapies alternative to stem cell-based approaches could be an option for clinical use.

The major underlying mechanism of MSC therapy is the effect of multiple cytokines released from the transplanted MSC in vivo. The idea was to collect cytokines from MSC cultures in vitro as conditioned media (CM) and use them for cell-free regenerative therapy in vivo. Some studies have reported that CM derived from various stem cells, such as MSC and SHED, show efficacy for acute organ damage ^20,21^, cardiac injury^22^, and spinal cord injury^23,24^. One of the proposed treatments is for acute myocardial infarction, in which a high concentration of hepatocyte growth factor (HGF) in SHED-CM plays an important role in inflammation and apoptosis suppression, protecting the heart from acute ischemic injury^22^. In addition, treatment with SHED-CM may be effective against spinal cord injury through the anti-inflammatory effects of MCP-1 and Siglec-1^23^. Therefore, treatment with CM from stem cells can be equivalent to the transplantation of stem cells, although its availability may be limited. An advantage of allogeneic CM is that it avoids immune-related problems and minimizes surgical invasiveness. However, one of the issues in using primary SHED-derived CM is the limitation of cell growth due to the primary cell, instability of supply of CM, and the use of raw materials containing by-products during cell culture. However, this can be resolved by cell immortalization and purification.

In this study, we investigated the effects of immortalized SHED-derived CM (IM-SHED-CM) and its purified components using various cell-based assays. We compared IM-SHED-CM and the purified components in terms of dehydrogenase activity, migration activity, and antioxidative stress activity and evaluated which purified component was more effective and had similar activity to the original IM-SHED-CM and SHED-CM.

## Materials and methods

### Materials

NIH3T3 (mouse fibroblast) was purchased from the JCRB Cell Bank (National Institute of Biomedical Innovation, Osaka, Japan). Human umbilical vein endothelial cells (HUVECs), human-adipose derived stem cells (also recognized as human MSCs), EBM^™^-2 endothelial cell basal medium, and adipose-derived stem cell basal medium with supplements were purchased from Lonza (Walkersville, MD, USA).

Antibiotic–antimycotic (100×), Dulbecco’s modified Eagle medium (DMEM), Hanks’ balanced salt solution, fetal bovine serum (FBS), 0.05% trypsin–EDTA (1×), TrypLE Express (phenol red), TrypLE select (no phenol red), trypan blue solution, micro BCA^™^ protein assay kit, goat anti-mouse IgG (H+L) secondary antibody conjugated with horseradish peroxidase (HRP), and goat anti-rabbit IgG (H+L) secondary antibody conjugated with HRP were purchased from Thermo Fisher Scientific (Waltham, MA, USA).

Stericup Quick Release-GP sterile vacuum filtration system (0.22 µm pore size), Millex-GV low protein binding durapore (PVDF) membrane (0.22 µm pore size), protease inhibitor cocktail, and bovine calf serum (CS) were purchased from Merck (Darmstadt, Germany). Activated charcoal powder was purchased from Nacalai Tesque (Kyoto, Japan).

Dulbecco’s phosphate-buffered saline (PBS, pH 7.4), RIPA buffer (50 mM Tris–HCl, pH 8.0, 150 mM sodium chloride, 0.5% w/v sodium deoxycholate, 0.1% w/v sodium dodecyl sulfate, 1.0% w/v NP-40 substitute), hydrogen peroxide (H_2_O_2_), Quick-CBB PLUS, collagenase, dispase skim milk, TBS-T solution, and 5-sulfosalicylic acid were purchased from FUJIFILM Wako Pure Chemical (Osaka, Japan).

Centrifugal ultrafiltration filter units (Vivaspin Turbo 15 membranes; MWCO: 30 kDa, 50 kDa, 100 kDa) were purchased from SARTORIUS (Göttingen, Germany).

The PD-10 column was purchased from Cytiva (Tokyo, Japan). The glucose assay kit-WST, cell counting kit-8 (for the WST assay), glucose uptake assay kit-green, and total glutathione quantification kit were purchased from Dojindo Laboratories (Kumamoto, Japan). Acrylamide solutions and powders, protein ladders and standards (Markers), and Clarity^™^ western ECL substrate were purchased from BioRad (Hercules, CA, USA). Phospho-p44/42 MAPK (Erk1/2) (Thr202/Tyr204) and phospho-Akt (Ser473) antibodies were purchased from cell signaling technology (Danvers, MA, USA). Monoclonal anti-beta-actin antibody was purchased from Sigma-Aldrich (St. Louis, MO, USA).

FITC-labeled mouse anti-human CD73, FITC-labeled mouse anti-human CD90, FITC-labeled mouse anti-human CD105, FITC-labeled mouse anti-human CD34, and FITC-labeled mouse anti-human CD45 were purchased from BD Biosciences (Franklin Lakes, NJ, USA).

Chlorhexidine solution was purchased from Yamazen Pharmaceutical Co., Ltd. (Osaka, Japan) and povidone-iodine solution was purchased from Iwaki Pharmaceutical Co., Ltd. (Tokyo, Japan). Human tumor necrosis factor (TNF)-α was purchased from PeproTech Inc (Cranbury, NJ, USA). Three glucose transporter (GLUT) inhibitors (WZB117, BAY-876, and KL-11743) were purchased from Selleck Chemicals (Houston, TX, USA).

### Preparation of stem cells from human exfoliated deciduous teeth (SHED) and immortalized SHED (IM-SHED)

SHED were isolated as previously described^23,25^. After disinfection of deciduous or extracted human deciduous teeth (from an 8 year-old healthy individual) with 5% chlorhexidine solution or 10% povidone-iodine solution, the coronal part was divided, and the pulp tissue was collected using a dental reamer. The pulp tissue was suspended in DMEM with 10% FBS, and incubated with 2 mg/mL collagenase and dispase for 1 h at 37 °C. The supernatant was removed by centrifugation (777×*g*, 5 min, 4 °C), and then the pulp cells were collected. The pulp cells were resuspended in DMEM supplemented with 10 wt% FBS and 1 wt% antibiotic–antimycotic and cultured at 37 °C under 5% CO_2_ and 95% air. The pulp cells were subcultured by treating with 0.05% trypsin/EDTA solution (37 °C, 5 min) for further experiments. These cells were termed as SHED. This study was approved by the Ethics Committee of U-Factor Co. (UFE-001).

SHED were then immortalized by transfection of the SV40 gene with the Lenti viral vector using Applied Biological Materials Inc. (Richmond, Canada) according to the manufacturer’s protocol. Cell immortalization was assessed by reverse transcriptase-polymerase chain reaction of the SV40 gene after five passages of culture. These cells were termed IM-SHED. IM-SHED was cultured in DMEM supplemented with 10 wt% FBS and 1 wt% antibiotic–antimycotic at 37 °C under 5% CO_2_ and 95% air, and were subcultured by treatment with 0.05% trypsin/EDTA solution (37 °C, 5 min).

### Flow cytometry analysis of SHED and IM-SHED

To examine the stem cell markers of SHED, IM-SHED, and hMSC, the cells were analyzed using flow cytometry. SHED and IM-SHED were collected by treatment with TrypLE Select (37 °C, 3 min) and treated with FITC-labeled antibodies (anti-human CD73, CD90, CD105, CD34, CD45) according to manufacturer’s instructions. Briefly, the collected cells (3 × 10^5^ cells) were incubated with FITC-labeled antibodies (anti-human CD73, CD90, CD105, CD34, CD45: 20 times dilution, 3 µg/mL, 20 times dilution, five times dilution, five times dilution, respectively) for 30 min at 4 °C, and then measured via flow cytometry (GALLIOS, Beckman Coulter, Inc., California, USA).

### Preparation of conditioned medium of SHED (SHED-CM) and IM-SHED (IM-SHED-CM)

For the preparation of SHED-CM and IM-SHED-CM, SHED and IM-SHED were used, for which the cell passage number was within eight times and 55 times, respectively. We used the same batch of SHED-CM and IM-SHED-CM collected from the SHED and IM-SHED. After the cell culture medium was exchanged with FBS-free DMEM, the cells were incubated for 48 h at 37 °C under 5% CO_2_ and 95% air. The cells were washed thrice with PBS before adding FBS-free DMEM. The culture medium was then collected and passed through a membrane filter (Stericup Quick Release-GP sterile vacuum filtration system) to prepare SHED-CM and IM-SHED-CM.

### Preparation of fractionated component from SHED-CM and IM-SHED-CM

SHED-CM and IM-SHED-CM were centrifuged thrice to remove cell debris and small particles: 180×*g*, 3 min, room temperature; 500×*g*, 30 min, 4 °C; and 2000×*g*, 30 min, 4 °C. Subsequently, the collected CM was passed through Millex-GV PVDF membrane (0.22 µm). First, the collected SHED-CM and IM-SHED-CM were transferred to centrifugal ultrafiltration filter unit (MWCO: 100 kD) and centrifuged (2000×*g*, 20 min, 4 °C). Second, the filtrate was transferred to centrifugal ultrafiltration filter unit (MWCO: 50 kD) and centrifuged (2000×*g*, 20 min, 4 °C). Finally, the filtrate was centrifuged (2000×*g*, 20 min, 4 °C) using centrifugal ultrafiltration filter unit (MWCO: 30 kD).

The concentrate on centrifugal ultrafiltration filter unit (MWCO: 100 kD) was mixed with PBS (10 mL) for washing and centrifuged (2000×*g*, 20 min, 4 °C). This process was repeated five times to prepare the fractionated components (> 100 kD).

The filtrate obtained from this process was mixed with the concentrate on centrifugal ultrafiltration filter unit (MWCO: 50 kD) and centrifuged (2000×*g*, 20 min, 4 °C) to prepare the fractionated component (50–100 kD). Then, the filtrate from this process was mixed with the concentrate on centrifugal ultrafiltration filter unit (MWCO: 30 kD) and centrifuged (2000×*g*, 20 min, 4 °C) to prepare the next fractionated component (30–50 kD). Finally, salt exchange of the filtrate was performed using a PD-10 column equilibrated with PBS to obtain the final fractionated component (< 30 kD). All concentrates were freeze-dried and dissolved in Milli-Q water for further experiments. SHED-CM, IM-SHED-CM, and the fractionated components were diluted with DMEM supplemented with 0.25 or 0.4 wt% CH-CS for NIH3T3-based studies and with EGM-2 culture medium for HUVEC-based studies in this paper. The concentration of total protein was determined by microBCA assay.

### Analysis of SHED-CM and IM-SHED-CM by sodium dodecyl-sulfate polyacrylamide gel electrophoresis (SDS–PAGE)

SHED-CM and IM-SHED-CM and the fractionated components were analyzed by native-PAGE (12.5%) and SDS–PAGE (12.5%) under non-reduced and reduced conditions, respectively. Native-PAGE and SDS–PAGE were performed at 10 mA for 190 min and 230 min, respectively. The proteins were visualized on a gel using Quick-CBB PLUS and Milli-Q water according to the manufacturer’s instructions.

### Liquid chromatography–mass spectrometry (LC–MS) analysis

SHED-CM and IM-SHED-CM and the fractionated components were analyzed using LC-MC by Proteobiologics Co., Ltd (Osaka, Japan) according to the manufacturer’s instructions using Q Exactive LC–MS combining UltiMate 3000 RSLCnanoHPLC with ReproSil-Pur 120 C18-AQ1.9 µm resin (Thermo Fisher Scientific).

### Measurement of ammonia and lactate concentration

Ammonia and lactic acid levels in the culture medium were analyzed by Hoken Kagaku, Inc. (Kanagawa, Japan). The concentrations of ammonia and lactic acid were determined using enzyme cycling and enzymatic methods, respectively.

### Measurement of dehydrogenase activity of cells exposed to SHED-CM, IM-SHED-CM, and the fractionated components using WST assay

Charcoal (10 mg) was mixed with CS (1 mL) by mild mixing for 15 s and incubated for 10 min at 37 °C. Then, the supernatant was collected after centrifugation (13,000×*g*, 20 min, 4 °C) and passed through Millex-GV PVDF membrane (0.22 µm) to prepare CH-CS, in which proteins were removed, and used as a negative control.

NIH3T3 cells were seeded into 96 well plate (5 × 10^3^ cells/well) and cultured in culture medium for 1 day. For starvation, the culture medium was replaced with DMEM supplemented with 0.4 wt% CH-CS for 24 h. SHED-CM, IM-SHED-CM, and fractionated components (> 100 kD, 50–100 kD, 30–50 kD, and < 30 kD) were diluted with DMEM supplemented with 0.4 wt% CH-CS, and were used for the NIH3T3 cell culture for 44 h. WST solution (10 µL) from cell counting kit-8 was added into each well and incubated for 4 h, and the absorbance was measured at 450 nm. For the inhibition assay, fractionated components (> 100 kD, 50–100 kD, 30–50 kD, and < 30 kD) were mixed and used for cell culture, followed by the WST assay.

HUVEC were seeded into 96 well plate (2.5 × 10^3^ cells/well) and cultured in EGM-2 culture medium for a day. HUVEC were then incubated with SHED-CM, IM-SHED-CM, and fractionated components (> 100 kD, 50–100 kD, 30–50 kD, and < 30 kD) diluted in EGM-2 for 3 days. WST solution (10 µL) from cell counting kit-8 was added into each well and incubated for 4 h and the absorbance was measured at 450 nm. For the inhibition assay, fractionated components (> 100 kD, 50–100 kD, 30–50 kD, and < 30 kD) were mixed and used for cell culture, followed by the WST assay.

### Analysis of intracellular signaling pathways by western blotting

NIH3T3 cells were seeded into 12 well (6 × 10^4^ cells/well) and cultured in culture medium containing 10 wt% CS for a day. For starvation, the culture medium was replaced with DMEM supplemented with 0.4 wt% CH-CS for 24 h.

SHED-CM, IM-SHED-CM, and fractionated components (> 100 kD, 50–100 kD, 30–50 kD, and < 30 kD) were diluted with DMEM supplemented with 0.4 wt% CH-CS, and were used for the NIH3T3 cell culture for 30 min. As a control, 10 wt% FBS and human TNF-α (50 ng/mL) were mixed with DMEM. After washing twice with PBS, the cells were lysed with RIPA buffer supplemented with a protease inhibitor cocktail (one tablet in 50 mL of RIPA buffer). The cell lysate was collected into a microtube and incubated for 20 min at 4 °C. After centrifugation (20,000×*g*, 20 min, 4 °C), the supernatant was collected. A sample buffer containing SDS (Laemmli sample buffer: 62.5 mM Tris–HCl, pH 6.8, 2% SDS, 25% glycerol, 0.01% bromo phenol blue) was mixed with the supernatant and incubated for 5 min at 95 °C, followed by electrophoresis via 10% SDS–PAGE (10 mA for 70 min, 20 mA for 1.5 h) using mini-slab (ATTO, Tokyo, Japan).

Protein samples were transferred to a nitrocellulose membrane (Merck) and blocked with 5 wt% skim milk in TBS-T solution (FUJIFILM Wako Pure Chemical) for 1 h at room temperature. The nitrocellulose membrane was then incubated in a solution of primary antibodies at 4 °C overnight. After washing three times with TBS-T at room temperature, the nitrocellulose membrane was incubated with a secondary antibody for 1 h at room temperature.

Here, we used anti-phospho-p44/42 MAPK (Erk1/2) (Thr202/Tyr204) antibody (1:2000 dilution), phospho-Akt (Ser473) antibody (1:2000 dilution) as primary antibodies and anti-beta-actin antibody (1:2000), and anti-rabbit IgG secondary antibody conjugated with HRP (1:5000 dilution) and anti-mouse IgG secondary antibody conjugated with HRP (1:5000 dilution) as secondary antibodies. The membrane was treated with Clarity^™^ western ECL substrate and imaged using a Chemi Doc XRS+ system (BioRad, Hercules, CA, USA).

### Scratch assay of NIH3T3 cells

NIH3T3 cells were seeded into 48 well plate (3.8 × 10^4^ cells/well) and cultured in DMEM supplemented with 10 wt% CS for a day. For starvation, the culture medium was replaced with DMEM supplemented with 0.4 wt% CH-CS for 24 h. A single line of scratch was made in the NIH3T3 cells monolayer using a 200 µL micropipette tip. After washing twice with DMEM, the following solutions were added: SHED-CM (two times diluted with 0.4 wt% CH-CS/DMEM), IM-SHED-CM (two times diluted with 0.4 wt% CH-CS/DMEM), and fractionated components (> 100 kD, 50–100 kD, 30–50 kD, and < 30 kD), which were diluted with DMEM supplemented with 0.4 wt% CH-CS. The final concentration of total protein was 1.5 µg/mL, 90 µg/mL, 0.37 µg/mL, and 133 µg/mL, respectively, for fractionated components, which was determined by microBCA assay. The treated cells were cultured for 24 h. The scratched area at 0 and 24 h was analyzed using ImageJ software (version 1.52o; NIH, Bethesda, MD, USA).

### Inhibition assay using GLUT inhibitors

NIH3T3 cells were seeded into 96 well plate (5.0 × 10^3^ cells/well) and cultured in DMEM supplemented with 10 wt% CS for a day. For starvation, the culture medium was replaced with DMEM supplemented with 0.4 wt% CH-CS for 24 h. Then, GLUT inhibitors (WZB117^26^, BAY-876^27^, KL-11743^28^, 50 mM stock solution in DMSO) diluted with DMEM with 0.4 wt% CH-CS were added into each well (100 µL). After the treated cells were cultured for 44 h, WST solution (10 µL) from cell counting kit-8 was added into each well and incubated for 4 h and the absorbance was measured at 450 nm.

### H_2_O_2_-induced cytotoxicity assay

HUVEC were seeded into 96 well plate (5.0 × 10^3^ cells/well) and cultured in EGM-2 culture medium for a day. HUVEC were then incubated with IM-SHED-CM and fractionated components (> 100 kD, 50–100 kD, and 30–50 kD) diluted in EGM-2 culture medium for 3 days. An aqueous solution of H_2_O_2_ was added into each well (final concentration: from 3.1 × 10^−4^ to 5.0 × 10^−3^%) and the cells were incubated for 1 h at 37 °C. After washing with EGM-2 culture medium, WST solution (10 µL) from cell counting kit-8 was added into each well and incubated for 3 h and the absorbance was measured at 450 nm.

### Glucose assay

To evaluate glucose metabolism, we measured the glucose concentration in the culture medium and studied glucose uptake in the cells. After HUVEC or HeLa cells were collected by treatment with TrypLE Express, the cells were seeded into 48 well plate (3.0 × 10^4^ cells/well) and cultured in each growth medium for a day. The cells were then incubated in IM-SHED-CM diluted in the culture medium for 3 days. The glucose concentration of the collected supernatant was measured using the glucose assay kit-WST, according to the manufacturer’s protocol.

We also measured glucose, which was taken up directly by the cells. After HeLa cells were collected by treatment with TrypLE Express, the cells were seeded into 96 well plate (4.0 × 10^4^ cells/well) and cultured in MEM with 10% FBS for a day. The cells were incubated in IM-SHED-CM diluted in culture medium for 1 day. The glucose uptake assay kit-green was used to measure glucose uptake according to the manufacturer’s protocol. Briefly, the culture medium was replaced with glucose- and serum-free MEM supplemented with fluorescently labelled glucose for 15 min. After washing with glucose and serum-free MEM, the fluorescence intensity of the cells was measured using a fluorescence microplate reader (Ex: 488 nm, Em: 520 nm, TECAN Infinite M200, Männedorf, Switzerland).

### Determination of total glutathione (GSH) concentration of cells after exposure to IM-SHED-CM

HUVEC were seeded into six well plate (1.5 × 10^5^ cells/well) and cultured in EGM-2 culture medium for 1 day. Then, HUVEC were incubated with IM-SHED-CM diluted with EGM-2 culture medium for 2 days (dilution ratio: from 0.39 to 50 wt%). After the cell culture, the HUVEC were treated with TrypLE^™^ Express and centrifuged (220 × *g*, 5 min, 4 °C) using Hanks’ balanced salt solution. The collected cells were mixed with 5-sulfosalicylic acid solution (20 µL) and centrifuged (8000×*g*, 10 min, 4 °C). The supernatant was diluted 10 times and the concentration of GSH was determined using a total GSH quantification kit according to the manufacturer’s protocol.

### Statistical analysis

All results were repeated at least thrice and are presented as means ± standard deviations. For statistical calculations, a one-way ANOVA was used followed by Dunnett’s multiple comparison test, which was performed using GraphPad Prism 8 for MacOS version 8.4.2 (GraphPad Software, La Jolla, CA, USA). The cutoff for statistical significance was p < 0.05, with the levels of significance indicated as follows: *p < 0.05, **p < 0.01, ***p < 0.001, and ****p < 0.0001.

## Results

### Characterization of CMs and fractionated components

**Figure 1:**
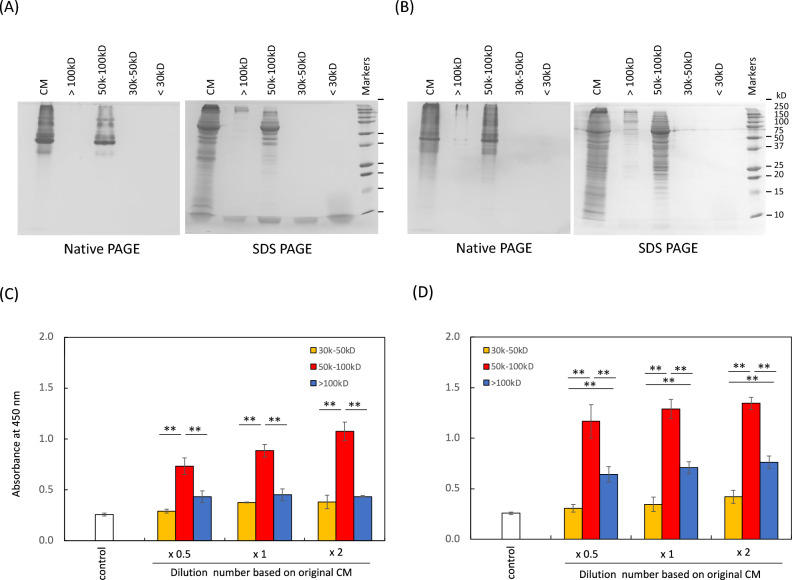
Representative image of Native-PAGE and SDS-PAGE gels with Coomassie Brilliant Blue staining of original CM and fractionated components (> 100, 50–100, and 30–50 kD) from (**A**) SHED-CM and (**B**) IM-SHED-CM together with a 10–250 kD ladder. Dehydrogenase activity of (**C**,**D**) NIH3T3 cells and fractionated components from (**C**) SHED-CM and (**D**) IM-SHED-CM (n = 3). CH-CS were used as a negative control. The original data of (**A**,**B**) was shown in Fig. S5.

Cells were isolated from exfoliated deciduous teeth, as previously described^9,23,25^, and the surface markers were analyzed using flow cytometry. The analysis showed that CD73, CD105, and CD90 were expressed, whereas CD45 and CD34 were not expressed on the cells (Supplementary Fig. S1), indicating that the isolated cells were SHED. The expression pattern of SHED surface markers was similar to that of adipose-derived MSC (Supplementary Fig. S1), again proving that the isolated cells were SHED^9^. In addition, IM-SHED showed a similar expression pattern of surface markers to that of SHED, indicating that the properties of the stem cells were well maintained after immortalization. The pattern of surface marker expression in IM-SHED was maintained until passage number 130, as examined by flow cytometry (Supplementary Fig. S1), indicating that it is possible to culture IM-SHED over a long period of time. Subsequently, SHED and IM-SHED were used to collect CM after 48 h of culture, which contained secreted components from the cells, such as cytokines and metabolites. In this study, CMs were purified by ultrafiltration into fractions of > 100, 50–100, 30–50, and < 30 kD. The results of Native-PAGE and SDS–PAGE of SHED-CM, IM-SHED-CM, and fractionated components are also shown (Fig. 1A, B). Some bands were derived from the protein components detected in SHED-CM and IM-SHED-CM. After purification of CMs by ultrafiltration into each fraction (> 100, 50–100 kD, 30–50, < 30 kD), most of the bands were detected in the > 100 and 50–100 kD fractions. LC–MS analysis revealed that approximately 50% of all kinds of proteins were present in 50–100 kD fraction from original IM-SHED-CM, which comprised cytokines such as VEGF, HGF, IL-6, TGF-beta, PDGF, and bFGF. The detection intensity from LC–MS was 2.6 × 10^6^, 2.3 × 10^5^, 1.4 × 10^6^, 1.9 × 10^6^, 4.3 × 10^4^, and 2.1 × 10^5^, for IM-SHED-CM, respectively and 5.0 × 10^6^, 1.6 × 10^5^, 1.4 × 10^5^, 3.3 × 10^5^, 2.3 × 10^4^, and 4.1 × 10^4^, for 50–100 kD fraction of IM-SHED-CM (arbitrary unit).

In addition, bands were not clearly visible in fraction of 30–50 kD and < 30 kD, however, some cytokines are actually present such as bFGF. Due to the limit of detection of CBB, they were not visible. There are bFGF and its variants, which were assess by WB (Supplementary Fig. S3).

### Dehydrogenase activity of cells exposed to CMs and fractionated components

To evaluate the effects of SHED-CM, IM-SHED-CM, and fractionated components on cellular activity, the intracellular dehydrogenase activity was studies using the WST assay. The WST assay is affected by dehydrogenase activity and/or the number of living cells. We can directly measure the intracellular dehydrogenase activity without destroying cells by WST, in which the resulting reductants were produced in the supernatant. There are a couple of assays for evaluation of cellular activities such as MTT assay. MTT assay can evaluate the cellular activity based on by the mitochondrial ability to metabolize MTT. The resulting products in the mitochondria were re-dissolved into organic solvent for the measurement. This assay is not direct measurement of the cellular activity because the resulting products were re-dissolved for the measurement. Therefore, we considered that direct measurement of cellular activities by WST was more accurate for the evaluation of effects of SHED-CM, IM-SHED-CM in this study.

The NIH3T3 cells were incubated with SHED-CM, IM-SHED-CM, and fractionated components to evaluate the cellular activity using the WST assay (Fig. 1C, D), and the cellular activity increased with an increase in absorbance at 450 nm. DMEM supplemented with 10% CS was used as a positive control. SHED-CM and IM-SHED-CM showed higher values of intracellular dehydrogenase and a higher activity than the control serum-free medium and lower activity than the serum-supplemented medium of the positive control. In addition, only the 50–100 kD fractionated component showed higher intracellular dehydrogenase activity than both > 100 and 30–50 kD fractionated components in the SHED-CM and IM-SHED-CM groups. This result indicated that SHED-CM, IM-SHED-CM, and 50–100 kD fraction contained components that can activate intracellular dehydrogenase activity. We also obtained results similar to those of HUVEC, in which the 50–100 kD fractionated component showed higher cellular activity (Supplementary Fig. S2A). In addition, there was no difference in the number of HUVEC after incubation with SHED-CM and IM-SHED-CM, except low cytotoxicity was observed at the highest concentration (Supplementary Fig. S2B, C).

**Figure 2:**
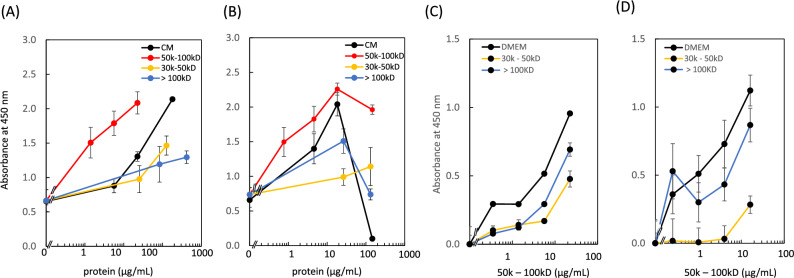
Comparison of dehydrogenase activity of NIH3T3 cells exposed to the same concentration of CMs and fractionated components (> 100, 50–100, and 30–50 kD) from (**A**) SHED-CM and (**B**) IM-SHED-CM (n = 3). Inhibition test for dehydrogenase activity of NIH3T3 by combining 50–100 kD fractionated components with > 100 or 30–50 kD fractionated component from (**C**) SHED-CM and (**D**) IM-SHED-CM (n = 3). The x-axis indicates the total protein concentration of 50–100 kD fractionated component. Total protein concentration was determined by microBCA assay. All the samples were diluted with CH-CS.

Based on these results, we further studied intracellular dehydrogenase activity by comparing each component with the same protein concentration for SHED-CM and IM-SHED-CM (Fig. 2A, B). Only the 50–100 kD fractionated component showed higher intracellular dehydrogenase activity than the original CMs. Similar results were obtained for SHED-CM and IM-SHED-CM. No higher activity was detected for > 100 and 30–50 kD fractionated components as compared to the original CMs. However, since the intracellular dehydrogenase activity was almost reduced to zero, all three fractionated components showed higher activity at the highest concentration (146 µg/mL) in IM-SHED-CM. We noticed that some metabolites such as ammonia and lactate were accumulated in CMs (ammonia = 130 µg/dL, lactate = 403 mg/dL in IM-SHED-CM) and this accumulation could reduce the intracellular dehydrogenase activity of NIH3T3 cell^29,30^. This negative effect was also observed in HUVEC exposed to IM-SHED-CM. However, the adverse effects of metabolites can be prevented by removing the related lower-molecular-weight components, resulting in the enhancement of intracellular dehydrogenase activity. In fact, the concentration of ammonia and lactate was 5 µg/dL and < 0.2 mg/dL, respectively, in the 50–100 kD fractionated component, indicating purification.

**Figure 3:**
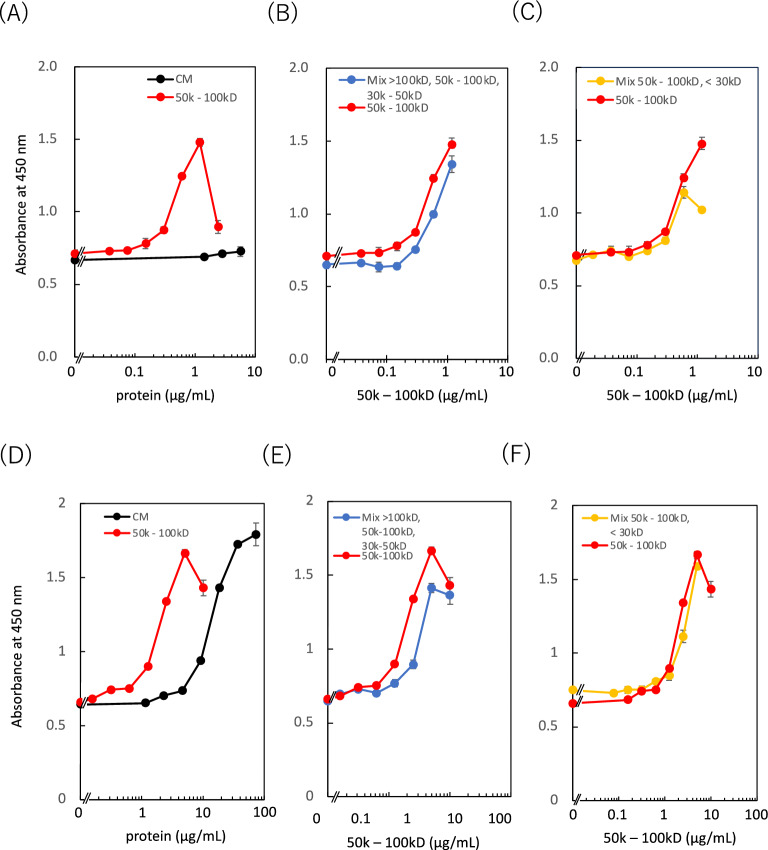
Comparison of dehydrogenase activity of HUVEC exposed to the same concentration of CMs and 50–100 kD fractionated component from (**A**–**C**) SHED-CM and (**D**–**F**) IM-SHED-CM (n = 3). Inhibition test for dehydrogenase activity of HUVEC by combining 50–100 kD fractionated component with a mixture of > 100, 50–100, and 30–50 kD fractionated components from (**B**) SHED-CM and (**E**) IM-SHED-CM (n = 3), and by combining 50–100 kD fractionated component with < 30 kD fractionated component from (**C**) SHED-CM and (**F**) IM-SHED-CM (n = 3). The x-axis indicates the total protein concentration of 50–100 kD fractionated component. Total protein concentration was determined by microBCA assay.

Subsequently, inhibition tests were performed to study the influence of the 50–100 kD fractionated component on cell activity. When the 50–100 kD fractionated component was mixed with > 100 or 30–50 kD fractionated components, intracellular dehydrogenase activity was reduced compared with the DMEM dilution (Fig. 2C, D). In particular, the degree of inhibition with the 30–50 kD fractionated component was higher than that with the serum-free medium. This indicates that some inhibitory factors were present in these two fractions, which could reduce intracellular dehydrogenase activity. This phenomenon was observed for both SHED-CM and IM-SHED-CM. Although we could not identify the inhibitory factors, they were different from the low-molecular-weight metabolites, and the molecular weight of the main components was assumed to be between 30–50 kD. Similar results were observed in HUVEC, where intracellular dehydrogenase activity was reduced when 50–100 kD fractionated component was mixed with > 100 and 30–50 kD or < 30 kD fractionated components from SHED-CM and IM-SHED-CM (Fig. 3). Presumably, some inhibitory factors were present in the two fractions, which could reduce intracellular dehydrogenase activity. Thus, it is possible to enhance intracellular dehydrogenase activity by purifying the 50–100 kDa fraction from the original CM in SHED-CM and IM-SHED-CM.

### Scratch assay using IM-SHED-CM and fractionated components

The scratch assay is an established method for quantitatively analyzing cell migration, which is strongly related to wound healing in vivo^31–34^. Skin wound healing is prompted by cytokines, chemokines, and growth factors secreted from stem cells such as MSC. Here, we performed the scratch assay using NIH3T3 cells to evaluate IM-SHED-CM and fractionated components. We studied the intracellular dehydrogenase activity of SHED-CM and IM-SHED-CM; however, only IM-SHED-CM was used for the scratch assay because there was no significant difference in the activity. In addition, we were able to obtain stable IM-SHED-CM because of the immortalized cell line, whereas primary SHED was not obtained. With an increase in the passage number of primary SHED, cell proliferation slowed and finally stopped at approximately passage 20. However, by immortalization, we overcame this issue to obtain stable IM-SHED-CM and found that the cell activity was quite similar to that of the original SHED-CM. Therefore, IM-SHED-CM was used in further studies.

**Figure 4:**
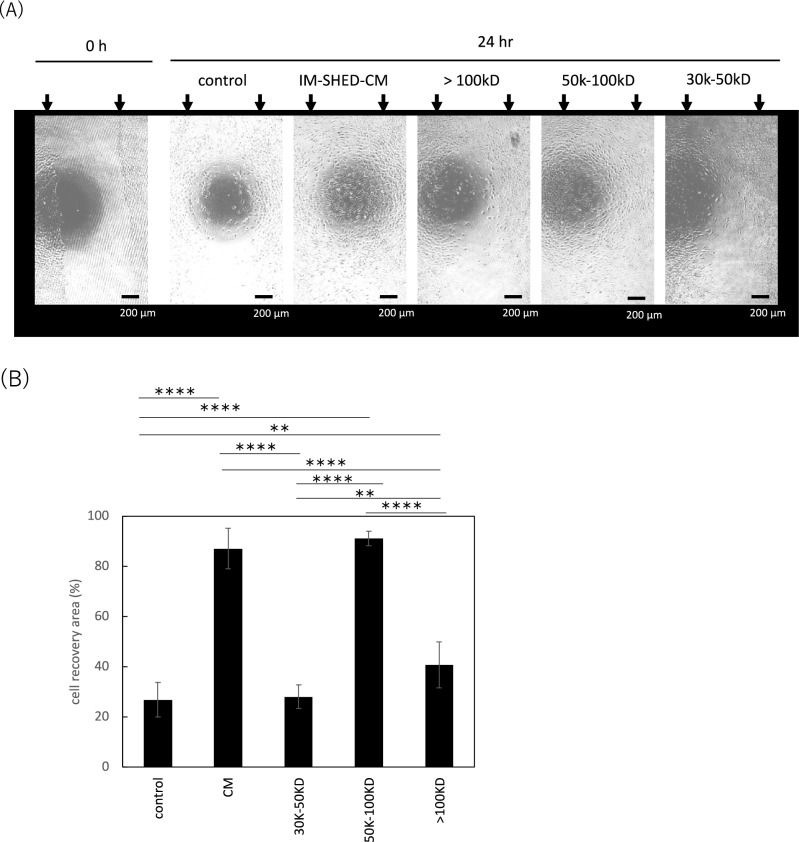
Scratch assay of NIH3T3 using IM-SHED-CM and fractionated components. (**A**) Phase contrast images taken at 0 h and 24 h after scratching. (**B**) Quantitative analysis of recovered area with migrating cells at 24 h (n = 3). CH-CS was used as a control. Arrows indicated the scracthed area.

To analyze cell migration quantitatively, we observed the exposed plastic surface after the cells were removed by scratching with a pipette tip for 24 h (Fig. 4). While approximately 55% of the exposed surface was covered with cells in serum-free medium in the control group, almost the entire plastic surface was covered with cells migrated from the IM-SHED-CM and 50–100 kD fractionated group. However, no difference was observed in recovery with cell migration between the control and > 100 and 30–50 kD fractionated component groups. This result indicates that the 50–100 kD fractionated component still maintained wound healing activity, which is similar to the original IM-SHED-CM. Although some studies have reported that CMs from primary MSCs have a wound healing effect^34^, there have been no reports on the wound healing effect of 50–100 kDa purified components from IM-SHED-CM. We considered that bFGF could mainly be involved in the cell migration effect because it was present in IM-SHED-CM (350 pg/mL) and the 50–100 kD fractionated component (16 pg/mL), as determined by ELISA and LC–MS. Due to the purification process via ultrafiltration, bFGF tended to reduce in the fractionated component. Presumably, this was because bFGF non-specifically attached to the ultrafiltration membrane like other proteins. The molecular weight of bFGF is approximately 16 kD. However, the variants were detected in proliferating cells, and they have functions similar to those of the original bFGF^35^. Several bFGF variants with higher molecular weights were detected in IM-SHED-CM and 50–100 kD fractionated component (Supplementary Fig. S3). Presumably, these variants in this fraction played an important role in migration activity, similar to that of the original IM-SHED-CM. In fact, when we ran the scratch assay of NIH3T3using recombinant bFGF (16 kD), the cell migration was observed (data not shown). However, the working concentration (1–10 ng/mL) in this assay was higher than that in IM-SHED-CM, indicating that the variants and/or other cytokines are more effective to the cell migration. Several cytokines, such as TGF-ß1, HGF, and EGF, can also promote cell migration^36^. TGF-ß1 and HGF were detected in IM-SHED-CM and 50–100 kD fractionated component; therefore, these cytokines also acted to promote cell migration^31,36^.

### Intracellular signaling by IM-SHED-CM and fractionated components

**Figure 5:**
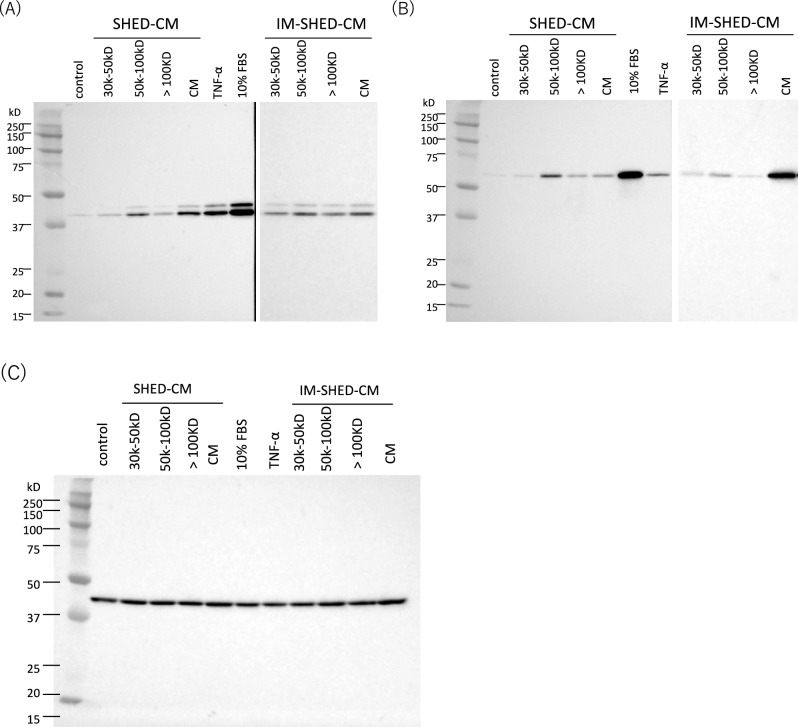
Western blot using specific antibodies for detecting phosphorylation of (**A**) MAPK, (**B**) Akt and (**C**) beta actin as a housekeeping protein in NIH3T3 after exposure to CMs and fractionated components (> 100, 50–100, and 30–50 kD) from SHED and IM-SHED-CM. CS and CH-CS were used as positive and negative controls, respectively. The original data of (**A**) and (**B**) was shown in Fig. S6 and Fig. S7, respectively.

To investigate intracellular signal transduction, the phosphorylation of MAPK and Akt was analyzed after the cells were treated with SHED-CM, IM-SHED-CM, and fractionated components. When cytokines such as bFGF are added to cells, the intracellular domain of receptor tyrosine kinases (RTKs) is transduced to activate the intracellular domain with phosphorylation, which follows the activation of MAPK and Akt^37–39^. Activation of MAPK and Akt via phosphorylation was detected when cells were treated with SHED-CM and IM-SHED-CM (Fig. 5). The same activation of MAPK and Akt was detected in cells treated with the 50–100 kD fractionated component under the same expression of housekeeping protein, beta actin (Fig. 5). From the quantitative analysis, the activation level of the 50–100 kD fractionated component from SHED-CM was 5.5 and 2.9 times higher than that of other two components (30–50 kD, > 100 kD) in MAPK. The similar results were obtained from Akt analysis (3.1 and 2.9 times higher). In addition, similar results were obtained from the 50–100 kD fractionated component of IM-SHED-CM; 1.6 and 1.4 times higher in MAPK and 2.2 and 3.1 times higher in Akt compared to other two components. This indicates that some cytokines can activate intracellular MAPK and Akt via signal transduction by RTK, which can activate cells. In particular, the 50–100 kD fractionated component in SHED-CM and IM-SHED-CM contained cytokines that directly interacted with RTK to activate intracellular signaling, resulting in cell migration ability. This signal transduction could also explain enhanced intracellular dehydrogenase activity.

### Cytotoxicity test against H_2_O_2_

**Figure 6:**
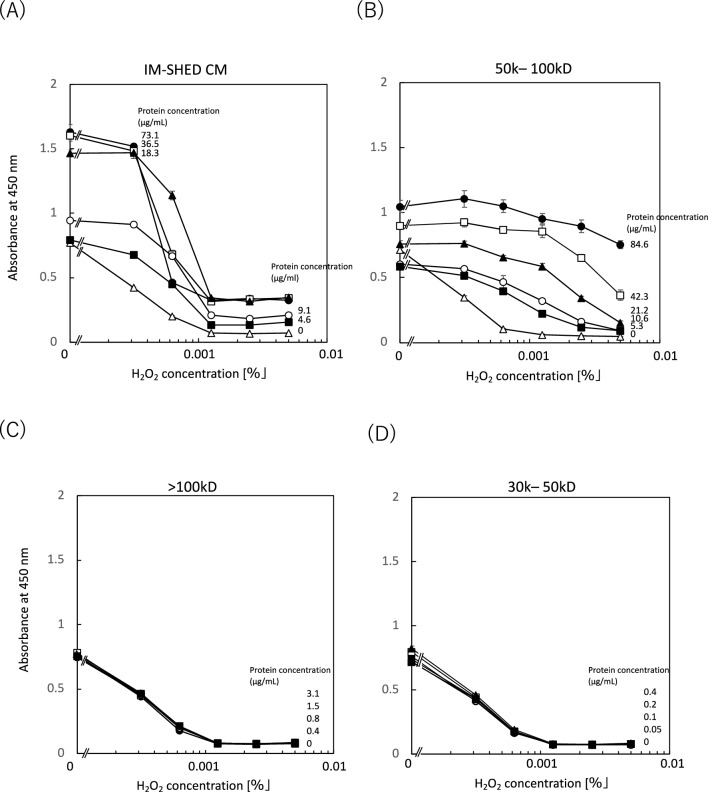
Hydrogen peroxide (H_2_O_2_)-induced oxidative stress test. After HUVEC were exposed to (**A**) IM-SHED-CM and fractionated components (**B**) > 100 kD, (**C)** 50–100 kD, (**D**) 30–50 kD, different concentrations of H_2_O_2_ were added, followed by the WST assay (n = 3). Total protein concentration was determined by microBCA assay.

A cytotoxicity test against H_2_O_2_ was performed to study the antioxidant activity of IM-SHED-CM and fractionated components, since MSCs can suppress oxidative stress^40,41^. We added different concentrations of H_2_O_2_ to HUVEC supplemented with IM-SHED-CM or fractionated components (Fig. 6). When HUVEC were cultured in EGM-2 medium (control group), followed by the addition of H_2_O_2_, the absorbance at 450 nm was lower than that of the other groups and decreased with an increase in H_2_O_2_ concentration, indicating that the cells were damaged by oxidative stress caused by H_2_O_2_. In contrast, when HUVEC were treated with IM-SHED-CM, the reduction in absorbance was suppressed compared to the control group, although the absorbance decreased with an increase in H_2_O_2_ concentration. This inhibitory effect was more pronounced with an increase in IM-SHED-CM concentration. Thus, IM-SHED-CM has a protective effect against antioxidative stress caused by H_2_O_2_. We then tested the fractionated components (> 100, 50–100, and 30–50 kD) (Fig. 6B–D). The 50–100 kD fractionated component showed an antioxidative effect, in which the reduction in absorbance was suppressed compared to that of the control group, although the absorbance decreased with an increase in H_2_O_2_ concentration. However, there was no antioxidative effect when > 100 and 30–50 kD fractionated components were added, in which all cells were damaged regardless of the addition of the two fractionated components.

**Figure 7:**
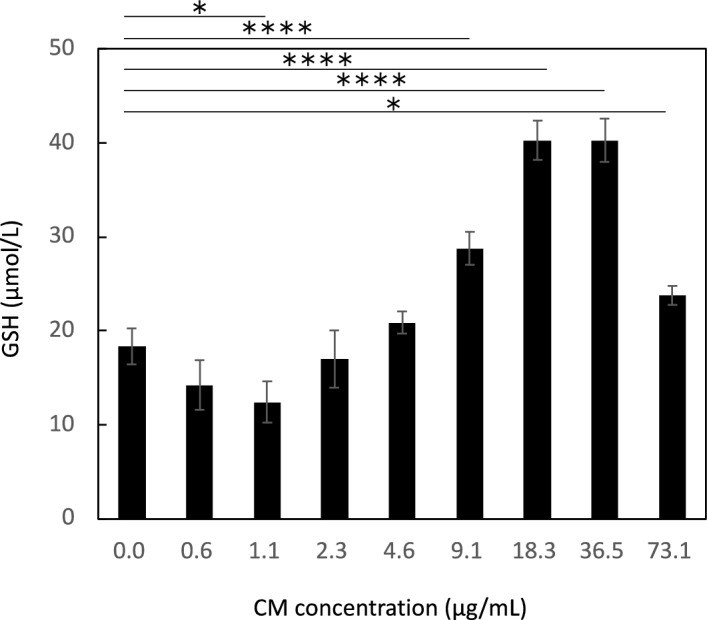
Determination of total glutathione concentration in cell. After HUVEC was incubated with IM-SHED-CM with different protein concentrations, the concentration of total intracellular glutathione was measured (n = 3).

To analyze the antioxidative activity of IM-SHED-CM and the 50–100 kD fractionated component, we focused on the GSH peroxidase and GSH reductase system, in which H_2_O_2_ is degraded enzymatically inside the cell. H_2_O_2_ is reduced to H_2_O by the oxidation of GSH to GSSG by GSH peroxidase, and GSH peroxidase is simultaneously reduced to GSH reductase. We determined the total concentrations of GSH and GSSG to evaluate the GSH peroxidase and GSH reductase systems as the enzymatic cycle depends on the concentration of substrates. In addition, we studied the cellular uptake of glucose, because the enzymatic cycle strongly depends on glucose consumption, as described in the next section. The total concentration of GSH in HUVECs was determined when the cells were mixed with different concentrations of IM-SHED-CM in EGM-2 medium (Fig. 7). The total GSH concentration increased in the IM-SHED-CM group compared to the control group, indicating that the enzymatic turnover of GSH peroxidase and reductase systems became more active to increase the apparent GSH concentration, resulting in the more rapid degradation of H_2_O_2_ for cell protection from oxidative stress. Therefore, the viability of HUVEC was improved by the addition of IM-SHED-CM and 50–100 kD fractionated component.

## Discussion

**Figure 8:**
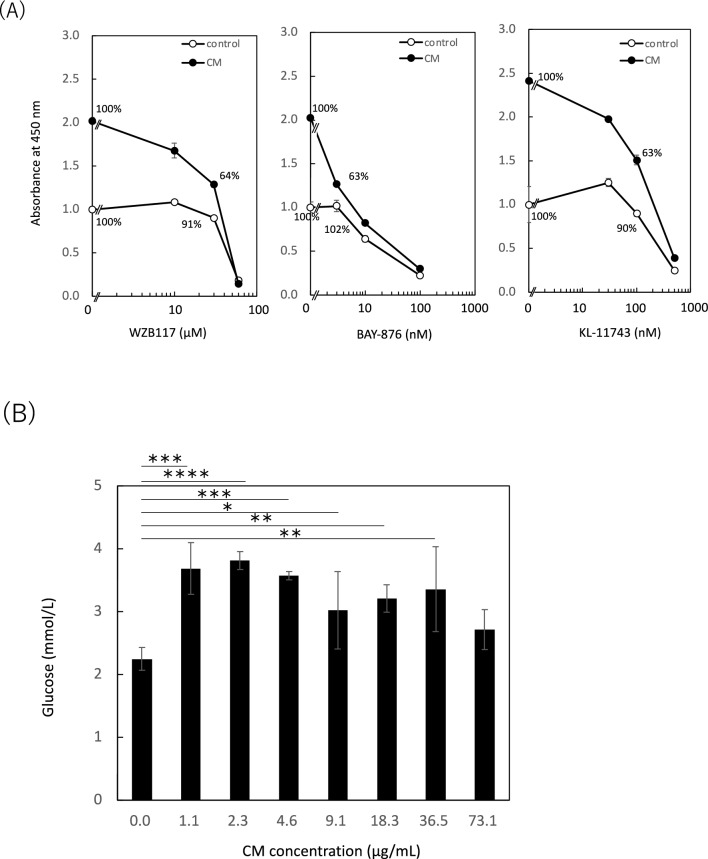
Influence of glucose uptake on cellular activity. (**A**) Inhibition assay for cellular dehydrogenase activity of NIH3T3 cells using GLUT inhibitors. After NIH3T3 cells were treated with three GLUT inhibitors (WZB117, BAY-876, KL-11743) and exposed to IM-SHED-CM, the WST assay was performed to compare the results with the inhibitor-free control group (n = 3). (**B**) Intracellular glucose uptake assay. After HUVEC were incubated with IM-SHED-CM with different protein concentrations, the intracellular glucose concentration was analyzed by direct measurement of the supernatant (n = 3).

Some cytokines stimulate glucose uptake via phosphatidylinositol-3 kinase/Akt activation of the GLUT^42,43^. We considered that some cytokines could activate intracellular signals to promote glucose uptake and the turnover of GSH peroxidase and reductase systems, which protect cells from oxidative stress. Therefore, we hypothesized that glucose uptake could be enhanced during cellular activation by IM-SHED-CM and 50–100 kD fractionated component. We studied the effect of glucose uptake on intracellular dehydrogenase activity by adding a GLUT inhibitor. Here, we used three different GLUT inhibitors: WZB117, BAY-876, and KL-11743, which are inhibitors of GLUT-1; GLUT-1, 2, 3, 4; and GLUT-1, 2, 3, respectively (Fig. 8A). When all three GLUT inhibitors were used in the WST assay, intracellular dehydrogenase activity decreased with increasing inhibitor concentration. However, the degree of reduction in absorbance at 450 nm differed between the serum-free medium (control) and IM-SHED-CM groups. When WZB117 was mixed with cells, the reduction in absorbance from 0 to 30 µM was approximately 9% and 40% for serum-free medium and IM-SHED-CM groups, respectively, indicating that the GLUT inhibitor influenced the intracellular dehydrogenase activity. Similar results were obtained when BAY-876 and KL-11743 were used, although the effective concentration differed owing to the different affinities of the inhibitors. The cell activity was reduced by approximately 40% from 0 to 3 µM and 0 to 100 µM, respectively, in the IM-SHED-CM group, whereas negligible or no reduction was observed in the control group. Thus, the reduction in cell activity by GLUT inhibitors was pronounced because glucose uptake was enhanced by the activation of IM-SHED-CM, and the influence of GLUT inhibitors increased, resulting in a large reduction in cell activity compared to the control group. We also analyzed the glucose concentration both in the culture medium using the enzymatic method and inside the cells using fluorescence labelled glucose to examine glucose uptake (Fig. 8B and Supplementary Fig. S4). When the concentration of glucose taken in the cells was calculated, more glucose was taken in the cells that were treated with IM-SHED-CM, and a maximum of 70% of the glucose uptake was significantly increased in the group treated with IM-SHED-CM. These results indicated that the uptake of glucose in cells was promoted by the addition of IM-SHED-CM. We considered that the enhanced glucose uptake could promote intracellular ATP production and enzymatic turnover of GSH peroxidase and reductase systems, resulting in the more rapid degradation of H_2_O_2_ to protect against cell damage. This also explains enhanced intracellular dehydrogenase activity.

Thus, we demonstrated accelerated glucose uptake via cytokine-stimulated GLUTs. One of the candidate cytokines was TGFß, which showed strong relationship with GLUT expression^44^ and IL-6^43^, which were detected in IM-SHED-CM and 50–100 kD fractionated component. Although IL-3, IL-1ß, and TNF-α were also reported to stimulate GLUT expression^42,43^, these cytokines were not detected in IM-SHED-CM.

Thus, the 50–100 kD fractionated components of IM-SHED-CM had an activity similar to that of the original CM from IM-SHED and SHED. For the purpose of the stable and large production as drugs, CM from IM-SHED was the only choice because primary SHED did not grow stably, and stopped the growth and prodcution of cytokines for the short term. Therefore, it would be beneficial to use the original CM from IM-SHED due to the lower cost than prufication. However, the original CM from IM-SHED showed strong cytotoxicty under higher concentration, which was not available as drugs. When we use 50–100 kD fractionated component from IM-SHED, we could completely overcome the problem. Therefore, we considered that the use of 50–100 kD fractionated component from CMs could be an alternative to cell transplantation of SHED and MSC due to their similar effects on cells.

## Conclusions

The 50–100 kD fractionated component from IM-SHED-CM had higher intracellular dehydrogenase activity than the original CMs, in which cell metabolites and lower molecular weight components could inhibit cell activity. The fractionated component also showed cell migration activity similar to that of the original IM-SHED-CM. In addition, the fractionated component showed a protective effect against oxidative stress when exposed to cells although the activity is slightly lower than the original CM. These higher activities could be explained by enhanced signal transduction and glucose uptake with some cytokines, such as bFGF, in the 50–100 kD fractionated component. Therefore, the use of 50–100 kD fractionated component could be an alternative therapy to stem cell transplantation, such as SHED and MSC, since purification from CM could improve efficacy.

### Supplementary Information

Supplementary Figures.

## Data Availability

The datasets used and/or analysed during the current study available from the corresponding author on reasonable request.
