# Supplementary material for: Activation of cellular antioxidative stress and migration activities by purified components from immortalized stem cells from human exfoliated deciduous teeth
Source: Sci Rep. 2024 Jul 3;14:15340. doi: 10.1038/s41598-024-66213-8 (PMC11222459; doi:10.1038/s41598-024-66213-8)
Supplement: Supplementary file 1 — Supplementary Figures. [file 41598_2024_66213_MOESM1_ESM.docx]

**Supplementary Information**

**Activation of cellular antioxidative stress and migration activities by purified components from immortalized stem cells from human exfoliated deciduous teeth**

Yujing Shu^1†^, Masato Otake^1†^, Yasuhiro Seta^2^, Keigo Hori^1^, Akiko Kuramochi^3^, Yoshio Ohba^3^, and Yuji Teramura^3,4,5^*

^1^U-Factor Co., Ltd., 4-8-1 Kojimachi Chiyoda, Tokyo 102-0083, Japan

^2^Smart Clinic Tokyo, K.PLAZA 2F, 1-7 Rokubancho, Chiyoda, Tokyo 102-0085, Japan

^3^Cellular and Molecular Biotechnology Research Institute (CMB), National Institute of Advanced Industrial Science and Technology (AIST), AIST Tsukuba Central 5, 1-1-1 Higashi, Tsukuba, Ibaraki 305-8565, Japan

^4^Department of Immunology, Genetics and Pathology (IGP), Uppsala University, Dag Hammarskjölds väg 20, SE-751 85, Uppsala, Sweden

^5^Master's/Doctoral Program in Life Science Innovation (T-LSI), University of Tsukuba, 1-1-1 Tennodai, Tsukuba, Ibaraki 305-8577 Japan

^†^**Equally contributed**

***Corresponding Author**

Yuji TERAMURA

Tel: +81(0)29-861-6582

E-mail: [y.teramura@aist.go.jp](mailto:y.teramura@aist.go.jp)

**Figure S1.** Flowcytometric analysis of hMSC, SHED (passage number 5 and 9), and IM-SHED (passage number 47 and 130) using antibodies against CD73, CD105, CD90, CD45, and CD34.

**Figure S2.** (A) Comparison of dehydrogenase activity of HUVEC exposed to IM-SHED-CM and fractionated components (> 100, 50-100, and 30-50 kD) (n = 3). Influence of CMs on cell number during HUVEC culture under (A) SHED-CM and (B) IM-SHED-CM.

**Figure S3.** Western blot using specific monoclonal antibodiy for detecting bFGF in IM-SHED-CM and fractionated components (> 100 and 50-100 kD).

**Figure S4.** Intracellular glucose uptake assay. After HeLa cells were incubated with IM-SHED-CM with different protein concentration, (A) the intracellular glucose concentration was analyzed by measuring fluorescence labelled glucose (n = 3) and (B) the supernatant was directly measured by enzymatic method (n = 3).

**Figure S5.** Original data of Fig.1 A and B. The gels of Fig.1 A and B were cropped from these gels.

**Figure S6.** Original data of Fig.5A. The gels of Fig.5A were cropped from these gels.

**Figure S7.** Original data of Fig.5B. The gels of Fig.5B were cropped from these gels.

**Figure S8.** Beta actin expression in SHED-CM and IM-SHED-CM for WB analysis in Fig. S7 and Fig. S8.

**Figure S9.** Original data of Fig.S3. The gels of Fig.S3 was cropped from the gels.
